# Supplementary figures and images for: Genome-Wide Analysis and the Expression Pattern of the MADS-Box Gene Family in Bletilla striata
Source: Plants (Basel). 2021 Oct 14;10(10):2184. doi: 10.3390/plants10102184 (PMC8539064; doi:10.3390/plants10102184)

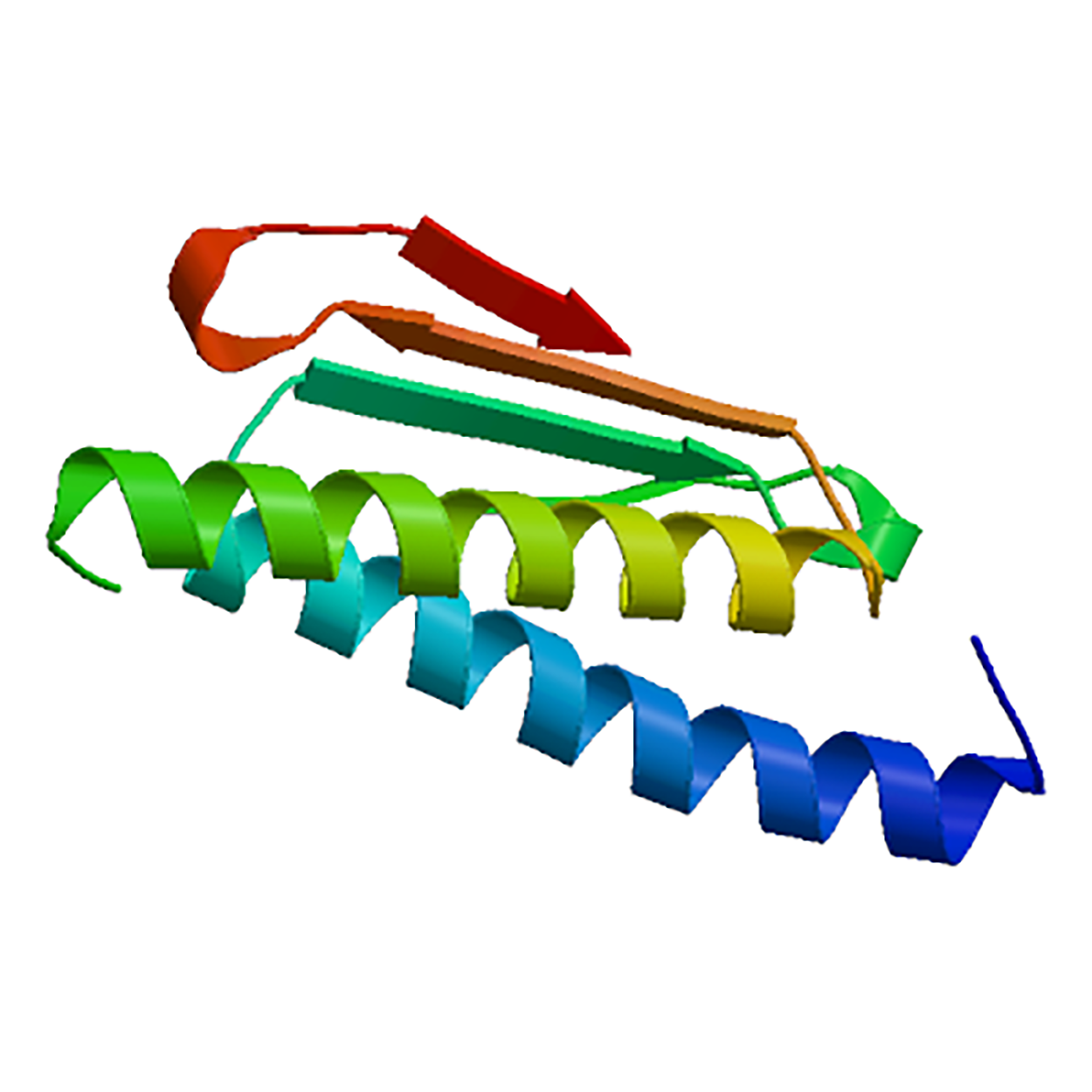

Supplement: Supplementary file 1 [file plants-10-02184-s001.zip › Figure S1. SRF-TF.tif]
